# Supplementary material for: Genome analysis of Parmales, the sister group of diatoms, reveals the evolutionary specialization of diatoms from phago-mixotrophs to photoautotrophs
Source: Commun Biol. 2023 Jul 7;6:697. doi: 10.1038/s42003-023-05002-x (PMC10328945; doi:10.1038/s42003-023-05002-x)
Supplement: Supplementary file 2 — Supplementary Information [file 42003_2023_5002_MOESM2_ESM.pdf]

Supplementary Information for:

Genome analysis of Parmales, the sister group of diatoms, reveals the evolutionary specialization of diatoms from phago-mixotrophs to photoautotrophs

Hiroki Ban[1], Shinya Sato[2], Shinya Yoshikawa[2], Kazumasa Yamada[2], Yoji Nakamura[3], Mutsuo Ichinomiya[4], Naoki Sato[5], Romain Blanc-Mathieu[1,6], Hisashi Endo[1], Akira Kuwata[7,*], Hiroyuki Ogata[1,*]

Corresponding author:

A. Kuwata, E-mail: akuwata@affrc.go.jp, Phone: +81-22-365-9929

H. Ogata, E-mail: ogata@kuicr.kyoto-u.ac.jp, Phone: +81-774-38-3270

This PDF file includes:

Supplementary Note

Supplementary Fig. 1 to 7

Captions for Supplementary Data 1 to 15

Supplementary Note

Comparison of the isolated genomes and Metagenome Assembled Genomes (MAGs)

To confirm the genomic features of paramleans identified in this study based isolate genomes, we examined two parmalean Metagenome Assembled Genomes (MAGs) from *Tara* Oceans Eukaryotic Genomes[1] (TARA_ARC_108_MAG_00221, TARA_PON_109_MAG_00217). Both MAGs were predicted as phago-mixotrophs by a gene-based phago-mixotrophy prediction model[2] (scores; 0.99 and 0.94 respectively). In addition, these MAGs had one silicic acid transporter (SIT) gene each (TARA_ARC_108_MAG_00221_000000002984.2.2, TARA_PON_109_MAG_00217_000000002599.19.1), as well as silicanin homologs (TARA_ARC_108_MAG_00221_000000001564.2.1, TARA_ARC_108_MAG_00221_000000002201.2.2, TARA_PON_109_MAG_00217_000000002307.2.1), which are missing from the bolidomonad (flagellate parmaleans) transcriptomes. These results indicate that the environmental parmaleans represented by the MAGs undergo phagocytosis and produce silicified cell walls. This is consistent with the observations obtained from the genome sequences of cultured members.

No phagotropy signals across diatoms.

We examined 54 diatom MAGs from *Tara* Oceans Eukaryotic Genomes[1] for phagotrophy signals. The prediction scores by a gene-based phago-mixotrophy prediction model[2] were consistently low (below 0.1). This result supports the idea that diatoms are universally not phagotrophic.

Some diatoms have N2-fixing cyanobacterial endosymbionts[3,4], or non-photosynthetic endosymbionts termed 'spheroid bodies'[5–7]. The origins of these endosymbionts are supposed to be recent, but how these endosymbionts entered the cell is unknown. To check whether these diatoms possess the capacity for phagotrophy, we examined a diatom with endosymbionts[4]. We downloaded the genome of *Epithemia pelagica* (GCA_946965045.1) from National Center for Biotechnology Information (NCBI). Genes were predicted by MetaEuk (v.4-a0f584d)[8] with default parameters using UniRef50[9] as the reference database. The quality of gene-prediction was checked by BUSCO (v.5.1.2)[10] with stramenopiles_odb10 dataset and the completeness was 98.0%. The predicted gene sequences were used as input for a gene-based phago-mixotrophy

prediction model[2]. The prediction score was 0.005, suggesting that the diatom with endosymbionts is not capable of phagocytosis. Some land plants like legumes, which do not have phagotrophy, have endosymbiotic bacteria internalized through endocytosis mechanisms (other than phagocytosis) within their root cells [11]. The internalization mechanism of the endosymbionts of diatoms may be similar to this.

No clear evidence of horizontal gene transfers in nitrogen metabolism genes

Previous studies have suggested that some of the genes involved in diatom nitrogen metabolisms, such as carbamate kinase and NADP(H) nitrite reductase, originated from bacteria through horizontal gene transfer (HGT)[12–14]. Such HGTs in diatoms may account for the absence of these genes in parmaleans. However, previous molecular phylogenetic analyses[15,16] indicate another possibility. The NAD(P)H nitrite reductase tree shows monophyly of diatoms and other ochrophytes, indicating that NAD(P)H nitrite reductase was vertically inherited by diatoms from ancestral ochrophytes but lost in parmaleans. In the cases of carbamate kinase, formamidase, cyanate lyase and hydroxylamine reductase, diatom sequences cluster with other eukaryotes, particularly dinoflagellates and haptophytes that contain secondary plastids derived from red algal endosymbionts, and Archaeplastida. This indicates the possibility that diatoms acquired these genes from their secondary symbiont and parmaleans lost them.

Carbon metabolism

Aquatic phototrophs, including diatoms and parmaleans, require CO_2 for photosynthesis. However, the concentration of CO_2 dissolved in water is much lower than the required concentration; therefore, they take up dissolved inorganic carbon (both CO_2 and HCO_3^-) from water and concentrate it in their cells to increase the CO_2 concentration around Rubisco and efficiently fix carbon[17]. These carbon concentrating mechanisms (CCMs) are called biophysical CCMs and are distinguished from biochemical CCMs, as found in terrestrial C4 plants. There are two main types of proteins involved in biophysical CCMs[18–20]: carbonic anhydrases (CA), of which there are several classes[21], and bicarbonate (HCO_3^-) transporters. In our results, each diatom genome contained about 20 CAs, whereas the parmalean genomes contained fewer than 10 CAs (Supplementary Fig. 6a). Of the seven classes of CAs present in the diatom genomes, the α- and ι-classes were absent from the parmalean genomes. By contrast, β-class CAs were present in all

parmaleans but absent in all diatoms except *Phaeodactylum tricornutum*. Regarding bicarbonate transporters, 7–15 genes were present in the diatom genomes, whereas 4–7 genes were present in the parmalean genomes (Supplementary Fig. 6b). Thus, diatoms have more genes involved in biophysical CCM than do parmaleans.

Glycolysis, gluconeogenesis, and pyruvate hub metabolism[22] are central pathways of carbon metabolism in diatoms. Previous studies have shown that diatoms have more genes involved in carbon metabolism than do green algae[22,23]. However, we found no significant differences in the number of genes involved in carbon metabolism and the predicted localization of gene products between diatoms and parmaleans (Supplementary Fig. 7). The mitochondrial pay-off phase of glycolysis, which is a known feature of diatom carbon metabolism, was also predicted to be present in parmaleans. Furthermore, this localized phase was also found in oomycetes and other ochrophytes in our datasets. Therefore, the mitochondrial pay-off phase is not specific to diatoms but common to stramenopiles, as already described in a previous work[24].

Fusion genes for TPI–GAPDH[22], which are the first enzymes of the diatom mitochondrial pay-off phase of glycolysis, were present in six parmalean genomes (missing in *Triparma laevis* f. *inornata* and *Triparma verrucosa*). Excluding *Tetraparma gracilis*, the fusion genes found in parmaleans target the mitochondria, as in diatoms. An additional GAPDH gene located upstream (and on the opposite strand) of the fusion gene conserved in most diatoms was also found in five parmalean genomes. This GAPDH gene was not found in the 'Scaly parma' and *Tetraparma gracilis* genomes, in which the TPI-GAPDH fusion gene was located at the end of the contig. It was previously suggested that this conserved gene order contributes to the coordinated regulation of the two genes using a bidirectional promoter and plays an important role at the starting point of glycolysis[22]. This characteristic gene order probably emerged in the common ancestor of diatoms and Parmales, as it was not found in other ochrophytes (*Aureococcus anophagefferens* and *Ectoculpus siliculosus*).

Diatoms use a prokaryote-like Entner–Doudoroff (ED) pathway for mitochondrial glycolysis[25]. In this pathway, 6-phosphogluconate dehydratase (EDD) and 2-keto-3-deoxyphosphogluconate aldolase (EDA) play major roles. Many stramenopiles possess EDD genes. By contrast, EDA genes are absent in stramenopiles except diatoms. Thus, a previous study suggested that the diatom EDA originated by HGT from bacteria[25]. In the present work,

parmaleans were found to possess both EDD and EDA genes (Supplementary Fig. 7). This result indicates that the prokaryote-like ED pathway is not specific to diatoms and was probably acquired from the diatom/Parmales common ancestor.

Supplementary References

1. Delmont, T. O. *et al.* Functional repertoire convergence of distantly related eukaryotic plankton lineages abundant in the sunlit ocean. *Cell Genomics* **2**, 100123 (2022).
2. Burns, J. A., Pittis, A. A. & Kim, E. Gene-based predictive models of trophic modes suggest Asgard archaea are not phagocytotic. *Nat. Ecol. Evol.* **2**, 697–704 (2018).
3. Caputo, A., Nylander, J. A. A. & Foster, R. A. The genetic diversity and evolution of diatom-diazotroph associations highlights traits favoring symbiont integration. *FEMS Microbiol. Lett.* **366**, fny297 (2019).
4. Schvarcz, C. R. *et al.* Overlooked and widespread pennate diatom-diazotroph symbioses in the sea. *Nat. Commun.* **13**, 799 (2022).
5. Prechtl, J., Kneip, C., Lockhart, P., Wenderoth, K. & Maier, U.-G. Intracellular Spheroid Bodies of Rhopalodia gibba Have Nitrogen-Fixing Apparatus of Cyanobacterial Origin. *Mol. Biol. Evol.* **21**, 1477–1481 (2004).
6. Kneip, C., Voβ, C., Lockhart, P. J. & Maier, U. G. The cyanobacterial endosymbiont of the unicellular algae Rhopalodia gibba shows reductive genome evolution. *BMC Evol. Biol.* **8**, 30 (2008).
7. Nakayama, T. *et al.* Spheroid bodies in rhopalodiacean diatoms were derived from a single endosymbiotic cyanobacterium. *J. Plant Res.* **124**, 93–97 (2011).

8. Levy Karin, E., Mirdita, M. & Söding, J. MetaEuk—sensitive, high-throughput gene discovery, and annotation for large-scale eukaryotic metagenomics. *Microbiome* **8**, 48 (2020).
9. Suzek, B. E. *et al.* UniRef clusters: a comprehensive and scalable alternative for improving sequence similarity searches. *Bioinformatics* **31**, 926–932 (2015).
10. Seppey, M., Manni, M. & Zdobnov, E. M. BUSCO: Assessing Genome Assembly and Annotation Completeness. in *Gene Prediction* (ed. Kollmar, M.) vol. 1962 227–245 (Springer New York, 2019).
11. Brewin, N. J. Plant Cell Wall Remodelling in the Rhizobium–Legume Symbiosis. *Crit. Rev. Plant Sci.* **23**, 293–316 (2004).
12. Bowler, C. *et al.* The Phaeodactylum genome reveals the evolutionary history of diatom genomes. *Nature* **456**, 239–244 (2008).
13. Bowler, C., Vardi, A. & Allen, A. E. Oceanographic and Biogeochemical Insights from Diatom Genomes. *Annu. Rev. Mar. Sci.* **2**, 333–365 (2010).
14. Vancaester, E., Depuydt, T., Osuna-Cruz, C. M. & Vandepoele, K. Comprehensive and Functional Analysis of Horizontal Gene Transfer Events in Diatoms. *Mol. Biol. Evol.* **37**, 3243–3257 (2020).
15. Smith, S. R. *et al.* Evolution and regulation of nitrogen flux through compartmentalized metabolic networks in a marine diatom. *Nat. Commun.* **10**, 4552 (2019).
16. Smith, S. *et al.* Phylogenies of nitrogen acquisition and metabolism genes in the model marine diatom Phaeodactylum tricornutum. 21521196 Bytes (2019) doi:10.6084/M9.FIGSHARE.6233198.

17. Reinfelder, J. R. Carbon Concentrating Mechanisms in Eukaryotic Marine Phytoplankton. *Annu. Rev. Mar. Sci.* **3**, 291–315 (2011).
18. Hopkinson, B. M., Dupont, C. L., Allen, A. E. & Morel, F. M. M. Efficiency of the CO2-concentrating mechanism of diatoms. *Proc. Natl. Acad. Sci.* **108**, 3830–3837 (2011).
19. Tsuji, Y., Nakajima, K. & Matsuda, Y. Molecular aspects of the biophysical CO2-concentrating mechanism and its regulation in marine diatoms. *J. Exp. Bot.* **68**, 3763–3772 (2017).
20. Schoefs, B., Hu, H. & Kroth, P. G. The peculiar carbon metabolism in diatoms. *Philos. Trans. R. Soc. B Biol. Sci.* **372**, 20160405 (2017).
21. Jensen, E. L., Maberly, S. C. & Gontero, B. Insights on the Functions and Ecophysiological Relevance of the Diverse Carbonic Anhydrases in Microalgae. *Int. J. Mol. Sci.* **21**, 2922 (2020).
22. Smith, S. R., Abbriano, R. M. & Hildebrand, M. Comparative analysis of diatom genomes reveals substantial differences in the organization of carbon partitioning pathways. *Algal Res.* **1**, 2–16 (2012).
23. Kroth, P. G. *et al.* A Model for Carbohydrate Metabolism in the Diatom Phaeodactylum tricornutum Deduced from Comparative Whole Genome Analysis. *PLoS ONE* **3**, e1426 (2008).
24. Río Bártulos, C. *et al.* Mitochondrial Glycolysis in a Major Lineage of Eukaryotes. *Genome Biol. Evol.* **10**, 2310–2325 (2018).
25. Fabris, M. *et al.* The metabolic blueprint of Phaeodactylum tricornutum reveals a eukaryotic Entner-Doudoroff glycolytic pathway: The Phaeodactylum tricornutum metabolic blueprint. *Plant J.* **70**, 1004–1014 (2012).

26. Fukasawa, Y. *et al.* MitoFates: Improved Prediction of Mitochondrial Targeting Sequences and Their Cleavage Sites*. *Mol. Cell. Proteomics* **14**, 1113–1126 (2015).
27. Almagro Armenteros, J. J. *et al.* Detecting sequence signals in targeting peptides using deep learning. *Life Sci. Alliance* **2**, e201900429 (2019).
28. Nielsen, H. Predicting Secretory Proteins with SignalP. in *Protein Function Prediction* (ed. Kihara, D.) vol. 1611 59–73 (Springer New York, 2017).
29. Gruber, A., Rocap, G., Kroth, P. G., Armbrust, E. V. & Mock, T. Plastid proteome prediction for diatoms and other algae with secondary plastids of the red lineage. *Plant J.* **81**, 519–528 (2015).
30. Matsui, M. & Iwasaki, W. Graph Splitting: A Graph-Based Approach for Superfamily-Scale Phylogenetic Tree Reconstruction. *Syst. Biol.* syz049 (2019) doi:10.1093/sysbio/syz049.

Supplementary Figures

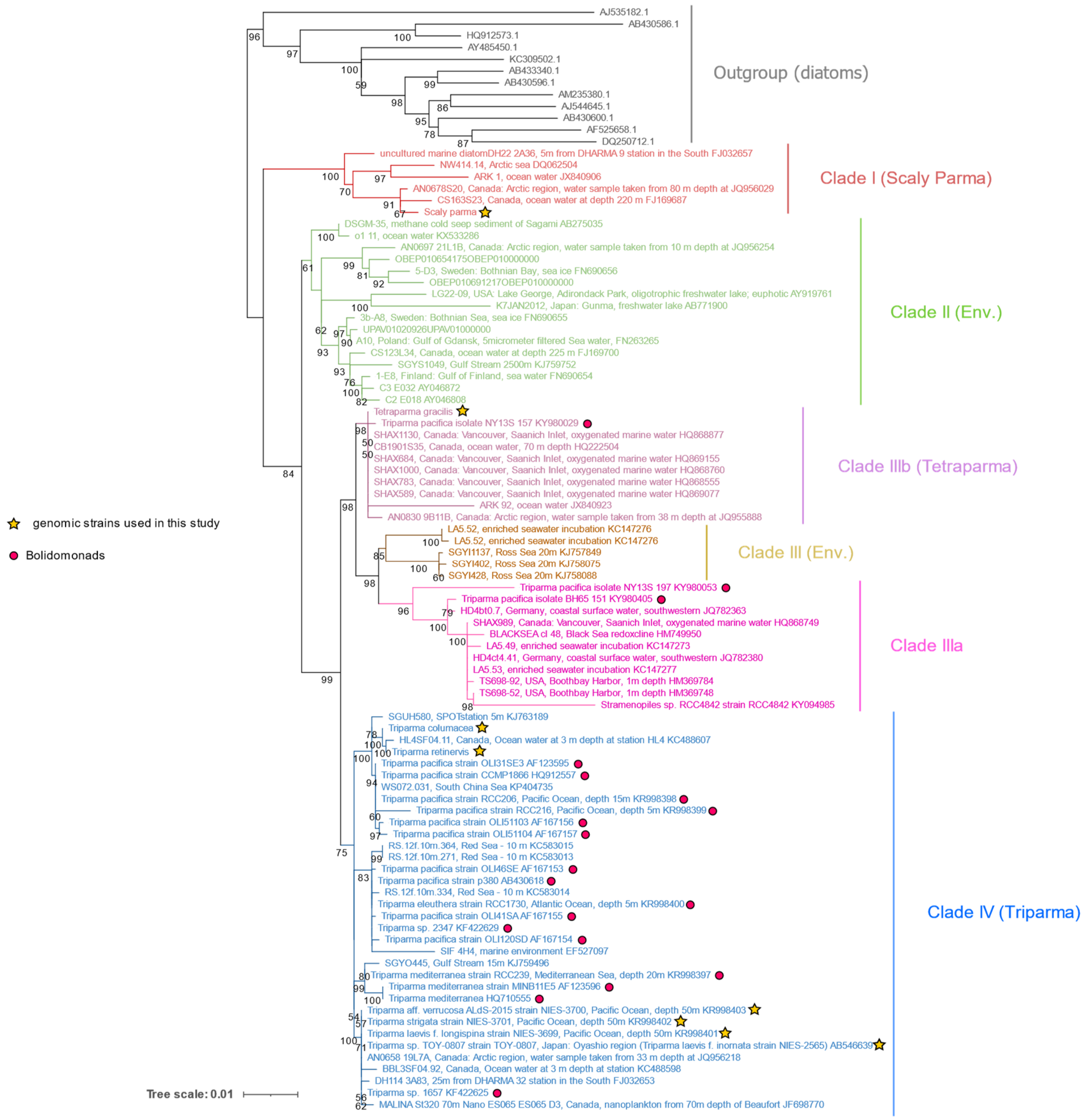

Supplementary Fig. 1 | Phylogenetic tree of 18 sRNA genes.

Maximum likelihood phylogenetic tree of the 18 sRNA genes of Parmales and diatoms (outgroup) from the SILVA database and our genomes. The tree was inferred by IQ-Trees2 and ultrafast bootstrap values > 50 were noted. Stars indicate the sequenced genomic strains, and red circles indicate isolated bolidomonad strains.

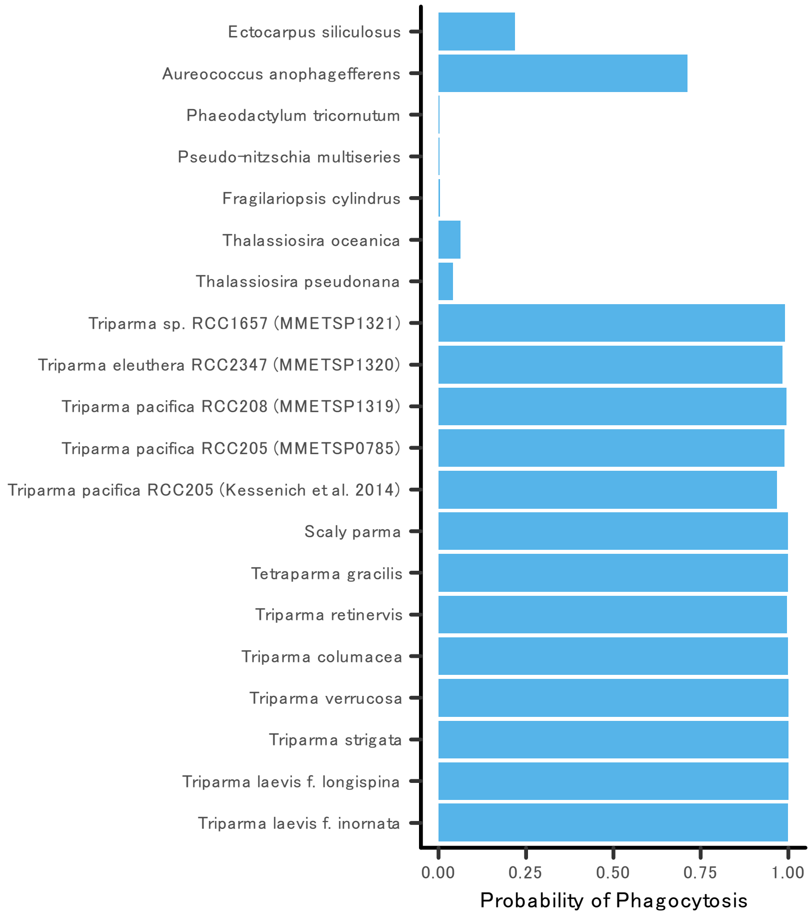

Supplementary Fig. 2 | Probability of phagotrophy calculated using a genome-scale tool developed by Burns et al. (2019). Source data are provided as Supplementary Data 6.

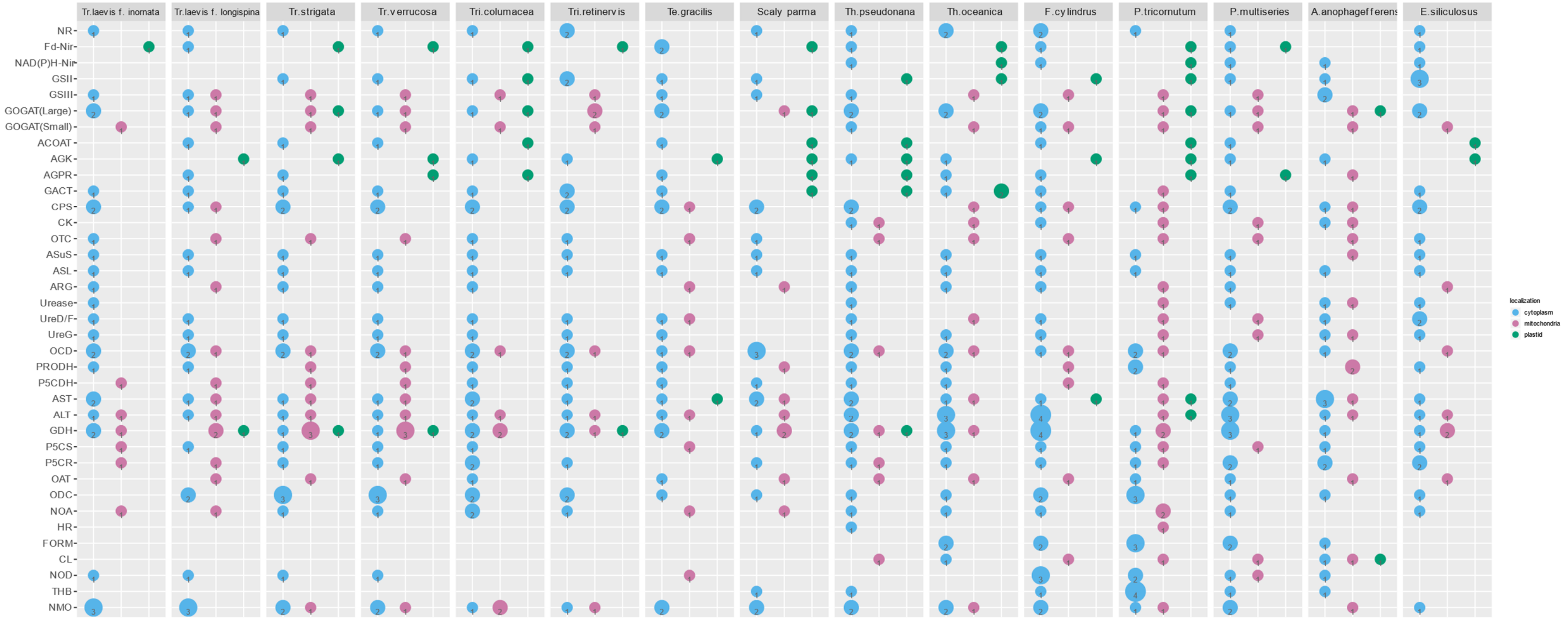

Supplementary Fig. 3 | Genes in nitrogen metabolism and their predicted intercellular localization.

Protein localization was predicted using MitoFates (v.1.1)[26], TargetP (v.2.0)[27], SignalP (v.4.1)[28], and ASAFIND (v.1.1.7)[29] and manually curated. Circle colours represent predicted intercellular localization. Pink: mitochondria; green: chloroplast; blue: cytoplasm. The size of the circle and the number below represent the number of associated genes. Gene names are abbreviated; full names and accessions can be found in Supplementary Data 9.

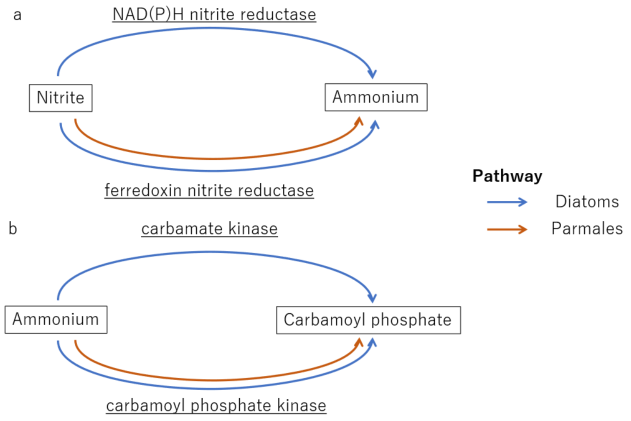

Supplementary Fig. 4 | Schematic view of pathways in N metabolisms.

Blue arrows show presence of the enzyme in diatoms and orange arrow shows that of parmaleans. (a) NAD(P)H nitrite reductase and ferredoxin nitrite reductase (b) carbamate kinase and carbamoyl phosphate synthetase.

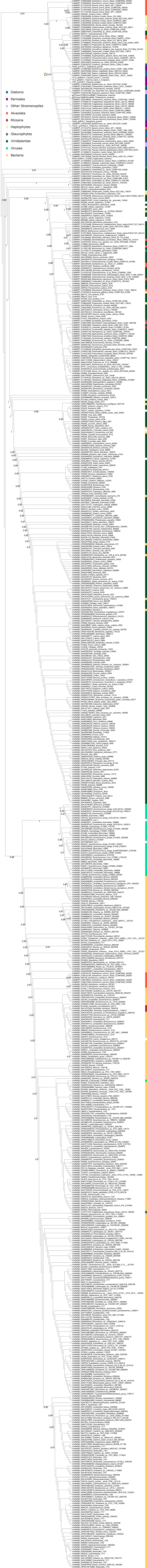
Diatoms
Parmales
Other Stramenopiles
Alveolata
Rhizaria
Haptophytes
Glaucophytes
Viridiplantae
Viruses
Bacteria

Supplementary Fig. 5 | Phylogenetic tree of plastocyanin genes of photosynthetic eukaryotes, cyanobacteria, and cyanophages.

The Graph Splitting method[30] was used to reconstruct the tree, with 100 replicates for the Edge Perturbation (EP) method for statistically evaluating branch reliability. Only EP scores > 0.9 are shown in the figure. The star represents the node supporting the monophyly of plastocyanin genes of diatoms and parmaleans (EP scores = 0.91).

a

taxa	α	β	γ	δ	ζ	θ	ι	Total
Pseudo–nitzschia multiseries	5	0	4	1	0	2	3	15
Phaeodactylum tricornutum	5	2	4	0	0	4	1	16
Fragilariopsis cylindrus	7	0	3	2	0	6	1	19
Thalassiosira oceanica	2	0	4	4	1	3	3	17
Thalassiosira pseudonana	3	0	4	4	1	5	1	18
Scaly parma	0	3	2	0	0	2	0	7
Tetraparma gracilis	0	3	2	0	0	1	0	6
Triparma retinervis	0	1	2	0	1	2	0	6
Triparma columacea	0	1	2	0	0	1	0	4
Triparma verrucosa	0	1	3	1	0	3	0	8
Triparma strigata	0	1	3	1	0	3	0	8
Triparma laevis f. longispina	0	1	3	2	0	3	0	9
Triparma laevis f. inornata	0	1	3	0	0	1	0	5
Ectocarpus siliculosus	1	2	3	0	0	0	0	6
Aureococcus anophagefferens	1	0	3	4	0	0	0	8

b

taxa	SLC4	SLC26
Pseudo–nitzschia multiseries	4	5
Phaeodactylum tricornutum	8	4
Fragilariopsis cylindrus	6	6
Thalassiosira oceanica	6	9
Thalassiosira pseudonana	3	4
Scaly parma	1	3
Tetraparma gracilis	3	3
Triparma retinervis	2	5
Triparma columacea	2	3
Triparma verrucosa	2	4
Triparma strigata	2	4
Triparma laevis f. longispina	2	4
Triparma laevis f. inornata	2	4
Ectocarpus siliculosus	2	2
Aureococcus anophagefferens	3	4

Supplementary Fig. 6 | Genes potentially involved in biophysical carbon concentration mechanisms (CCMs).

(a) Number of carbonic anhydrase genes potentially involved in biophysical carbon concentration mechanisms (CCMs). Greek letters (α, β, γ, δ, ζ, θ, ι) indicate the gene families (classes)[21]. Accessions can be found in Supplementary Data 11. (b) Number of bicarbonate transporter genes potentially involved in biophysical CCMs. Accessions can be found in Supplementary Data 12.

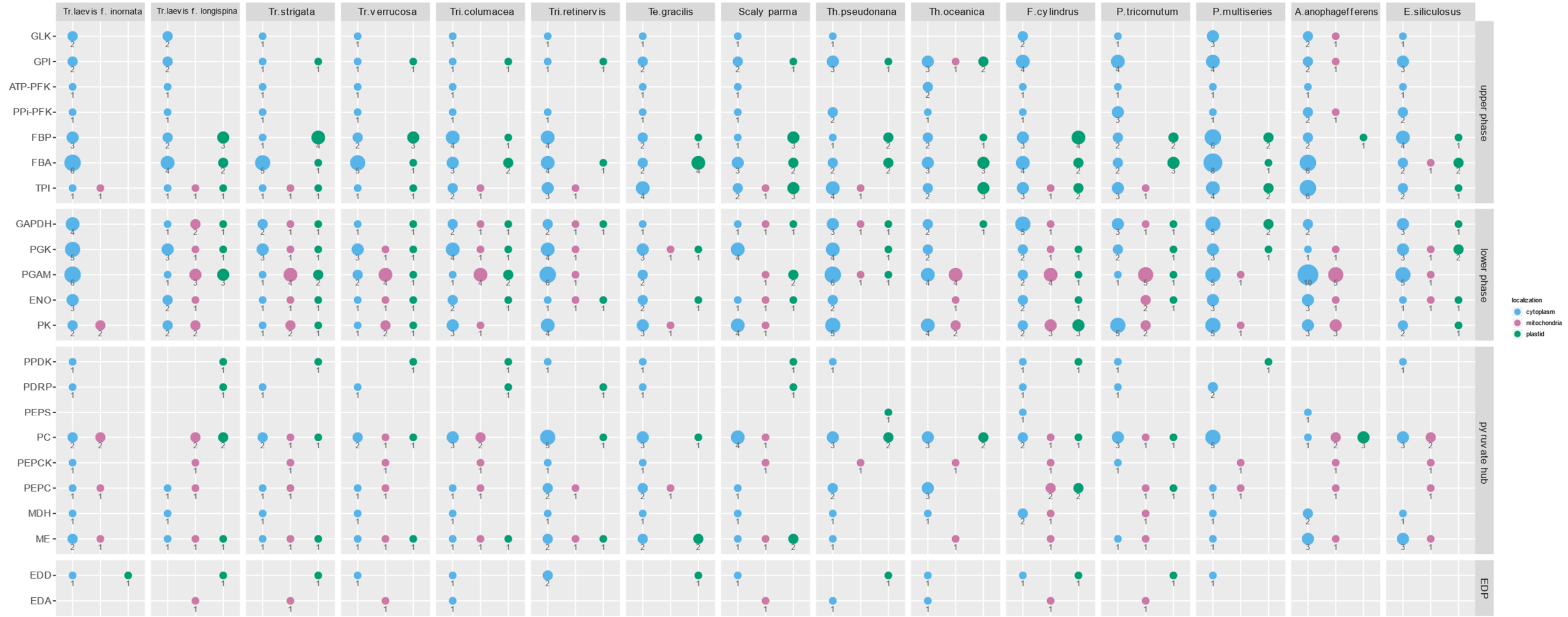

Supplementary Fig. 7 | Genes in carbon metabolism and their predicted intercellular localization.

Protein localization was predicted using MitoFates (v.1.1)[26], TargetP (v.2.0)[27], SignalP (v.4.1)[28], and ASAFIND (v.1.1.7)[29] and manually curated. Circle colours represent predicted intercellular localization. Pink: mitochondria; green: chloroplast; blue: cytoplasm. The size of the circle and the number below represent the number of associated genes. Gene names are abbreviated; full names and accessions can be found in Supplementary Data 13.

Description of supplementary files

Supplementary Data 1

The number of genes in different categories of orthologous groups. Taxonomic name(column A), Genes in core orthologs(B), Genes in shared orthologs(C), Genes in diatom-parmales shared orthologs(D), Genes in diatom specific orthologs (E), Genes in parmales specific orthologs (F) Genes in species unique orthologs (G).

Supplementary Data 2

InterPro domains enriched in diatom genomes. InterPro domain IDs (column A), InterPro domain description (B), Number of genes (C-O), the total number of genes annotated with this domain (P,Q), the total number of genes not annotated with this domain (R,S), *p*-values for Fisher's exact test (T), corrected *p*-values for multiple comparisons by Bonferroni correction (U) and manually assigned categories for InterPro domains (V).

Supplementary Data 3

InterPro domains enriched in parmalean genomes. Descriptions for column are same with Supplementary Data 2.

Supplementary Data 4

InterPro domains for START protein genes. InterPro domain IDs (column A), InterPro domain description (B), Number of genes (C-Q), the total number of genes annotated with this domain in parmalean genomes (R).

Supplementary Data 5

The number of calcium-binding protein genes. Taxonomic name (column A), number of genes (B), group names they belong to (C).

Supplementary Data 6

Probability of phagotrophy calculated using a genome-scale tool developed by Burns et al. (2019). Taxonomic name (column A), predicted probability of phagotrophy (B), group names they belong to (C).

Supplementary Data 7

Intraflagellar transport (IFT) subunits gene/transcripts catalog. Gene accessions (column A), gene name (B), InterPro ID / EggNOG ID used to estimate gene function (C), gene category (D) taxonomic name (E), and group names they belong to (F).

Supplementary Data 8

Transporter gene catalog. Gene accessions (column A), gene name abbreviation (B), gene name (C), InterPro ID / EggNOG ID used to estimate gene function (D), taxonomic name (E), and group names they belong to (F).

Supplementary Data 9

Nitrogen metabolism gene catalog. Gene accessions (column A), gene name abbreviation (B), gene name (C), InterPro ID / EggNOG ID used to estimate gene function (D), taxonomic name (E), group names they belong to (F), assigned subcellular localization based on targeting predictions (G-L), finally assigned subcellular localization (M), taxonomic name (N) and group names they belong to (O).

Supplementary Data 10

Iron metabolism gene catalog. Gene accessions (column A), gene name abbreviation (B), gene name (C), InterPro ID / EggNOG ID used to estimate gene function (D), taxonomic name (E), and group names they belong to (F).

Supplementary Data 11

Carbonic anhydrase gene catalog. Gene accessions (column A), gene name (B), InterPro ID / EggNOG ID used to estimate gene function (C), taxonomic name (D), and group names they belong to (E).

Supplementary Data 12

Bicarbonate transpoter gene catalog. Gene accessions (column A), gene name (B), InterPro ID / EggNOG ID used to estimate gene function (C), taxonomic name (D), and group names they belong to (E).

Supplementary Data 13

Carbon metabolism gene catalog. Gene accessions (column A), gene name abbreviation (B), gene name(C), pathway name (D), InterPro ID / EggNOG ID used to estimate gene function (E), taxonomic name (F), group names they belong to (G), assigned subcellular localization based on targeting predictions (H-M), finally assigned subcellular localization (N), taxonomic name (O) and group names they belong to (P).

Supplementary Data 14

Transcriptome data used in orthologous genes (OGs) clustering.

Supplementary Data 15

Genome data source we used in this study.
