# Supplementary material for: Genome analysis of Parmales, the sister group of diatoms, reveals the evolutionary specialization of diatoms from phago-mixotrophs to photoautotrophs
Source: Commun Biol. 2023 Jul 7;6:697. doi: 10.1038/s42003-023-05002-x (PMC10328945; doi:10.1038/s42003-023-05002-x)
Supplement: Supplementary file 3 — Description of Additional Supplementary Files [file 42003_2023_5002_MOESM3_ESM.pdf]

Description of Additional Supplementary Files

File name: Supplementary Data 1-14

Description: Numerical data supporting the figures.
