# Supplementary material for: Genome analysis of Parmales, the sister group of diatoms, reveals the evolutionary specialization of diatoms from phago-mixotrophs to photoautotrophs
Source: Commun Biol. 2023 Jul 7;6:697. doi: 10.1038/s42003-023-05002-x (PMC10328945; doi:10.1038/s42003-023-05002-x)
Supplement: Supplementary file 5 — Reporting Summary [file 42003_2023_5002_MOESM5_ESM.pdf]

nature portfolio

Corresponding author(s): Hiroyuki Ogata, Akira Kuwata

Last updated by author(s): Jun 25, 2023

Reporting Summary

Nature Portfolio wishes to improve the reproducibility of the work that we publish. This form provides structure for consistency and transparency in reporting. For further information on Nature Portfolio policies, see our Editorial Policies and the Editorial Policy Checklist.

Statistics

For all statistical analyses, confirm that the following items are present in the figure legend, table legend, main text, or Methods section.

n/a	Confirmed	
☐	☒	The exact sample size (n) for each experimental group/condition, given as a discrete number and unit of measurement
☒	☐	A statement on whether measurements were taken from distinct samples or whether the same sample was measured repeatedly
☐	☒	The statistical test(s) used AND whether they are one- or two-sided<br>*Only common tests should be described solely by name; describe more complex techniques in the Methods section.*
☒	☐	A description of all covariates tested
☐	☒	A description of any assumptions or corrections, such as tests of normality and adjustment for multiple comparisons
☒	☐	A full description of the statistical parameters including central tendency (e.g. means) or other basic estimates (e.g. regression coefficient) AND variation (e.g. standard deviation) or associated estimates of uncertainty (e.g. confidence intervals)
☐	☒	For null hypothesis testing, the test statistic (e.g. F, t, r) with confidence intervals, effect sizes, degrees of freedom and P value noted<br>*Give P values as exact values whenever suitable.*
☒	☐	For Bayesian analysis, information on the choice of priors and Markov chain Monte Carlo settings
☒	☐	For hierarchical and complex designs, identification of the appropriate level for tests and full reporting of outcomes
☒	☐	Estimates of effect sizes (e.g. Cohen's d, Pearson's r), indicating how they were calculated

Our web collection on statistics for biologists contains articles on many of the points above.

Software and code

Policy information about availability of computer code

Data collection	No software was used
Data analysis	perl v.5.18.4; Python v 2.7.11 and v3.6.1; R v3.6.1 (tidyverse v1.2.1); Trimmomatic v.0.38; Platanus v.1.2.4; BWA v.0.7.17; cgat v.0.2.6; GeneMarkS v4.30; DIAMOND v0.9.18; blast+ v2.11.0; Bernap v.0.6; tRNA-scan-SE v.1.23; AUGUSTUS v3.2.2; fastx-toolkit; v.0.0.13; Tophat v2.1.1; Cufflinks v2.2.1; Trinity v.2.0.6; blast+ v.2.2.29 Exonoerate. v.2.4.0; tRNAscan-SE v.2.0.7; infernal v.1.1.3; RepeatModeler v.2.0.1; RepeatMasker v.4.1.0; BRAKER2; STAR v.2.7.3a; InterProScan v.5.26-65.0; eggNOG-Mapper v.2.0.1; MitoFates v.1.1; TargetP v.2.0; SignalP v.4.1; ASAFIND v.1.1.7; OrthoFinder v.2.3.7; MAFFT v7.453; trimAl v.1.4.1; RAxML v.8.2.12; CD-HIT v.4.8.1; TransDecoder v.5.5.0; HMMER v.3.3.2 and v.3.4.2; gs2 v.2.4<br>Trophic mode prediction tool provided by Burns et al., 2018.

For manuscripts utilizing custom algorithms or software that are central to the research but not yet described in published literature, software must be made available to editors and reviewers. We strongly encourage code deposition in a community repository (e.g. GitHub). See the Nature Portfolio guidelines for submitting code & software for further information.

Data

Policy information about availability of data

All manuscripts must include a data availability statement. This statement should provide the following information, where applicable:

- Accession codes, unique identifiers, or web links for publicly available datasets
- A description of any restrictions on data availability
- For clinical datasets or third party data, please ensure that the statement adheres to our policy

Sequence data generated during the current study are available in DDBJ bioprojects, under accession number PRJDB14101 (RNA reads for Triparma laevis f. inornata), PRJDB13844 (DNA reads for the other seven strains), and PRJDB13933 (RNA reads for other strains). The assembly data analysed during the current study are also available in the DDBJ repository, under accession numbers BLQM01000001-BLQM01000902 (Triparma laevis f. inornata), BRXW01000001-BRXW01001055 (Triparma laevis f. longispina), BRXX01000001-BRXX01000659 (Triparma verrucosa), BRXY01000001-BRXY01000634 (Triparma strigata), BRXZ01000001-BRXZ01008760 (Triparma retinervis), BRYA01000001-BRYA01001858 (Triparma colmacea), BRYB01000001-BRYB01007082 (Tetraparma gracilis), and BRYC01000001-BRYC01001921 ('Scaly parma'). Data underlying Figs. and Supplementary Figs. are provided as Supplementary Data files or are available in GenomeNet FTP (https://www.genome.jp/).

Human research participants

Policy information about studies involving human research participants and Sex and Gender in Research.

Reporting on sex and gender	NA
Population characteristics	NA
Recruitment	NA
Ethics oversight	NA

Note that full information on the approval of the study protocol must also be provided in the manuscript.

Field-specific reporting

Please select the one below that is the best fit for your research. If you are not sure, read the appropriate sections before making your selection.

☐ Life sciences ☐ Behavioural & social sciences ☒ Ecological, evolutionary & environmental sciences

For a reference copy of the document with all sections, see nature.com/documents/nr-reporting-summary-flat.pdf

Ecological, evolutionary & environmental sciences study design

All studies must disclose on these points even when the disclosure is negative.

Study description	We investigate eight parmales genomes (one of them was already sequenced but annotated in this study; the other seven were newly sequenced and annotated) and five diatom publicly available genomes. We firstly compared differentially enriched protein domains between two organism groups using Fisher's exact test. We found a part of protein domains enriched in parmalean genomes are related to phagotrophy. So we applied the statistical model provided by Burns et al., 2018 to predict trophic mode using genomic information. Then we moved to more focused investigation of genes in specific pathways and functions. We also conducted phylogenetic analysis of silicon transporter genes and plastocyanin genes to gain insight into the relationships between their evolutionary patterns and the lifestyles of the two groups.
Research sample	For parmaleans, we used Triparma laevis f. inornata (NEIS-2656), Triparma laevis f. longispina (NIES-3699), Triparma verrucosa (NIES-3700), and Triparma strigata (NIES-3701), isolated from the Oyashio region of the western North Pacific. For other parmalean strains, water samples were collected at 10 m in the Notoro-ko lagoon (44º3'2.1" N, 144º9'38.8" E, December 2015) for Triparma retinervis, at 10 m in the Sea of Okhotsk (45º25'0" N, 145º10'0" E, June 2017) for Tetraparma gracilis and Triparma columacea, and at 30 m in the Sea of Okhotsk (44º30'0" N, 144º20'0" E, June 2014) for the uncharacterized 'Scaly parma'. The strains were isolated by serial dilution with siliceous cell wall labelling techniques described previously (Ichinomiya et al., 2011). The strains were cultured in f/2 medium at 5 °C under a light intensity of ca. 30 µmol photons m−2 s−1 (14:10 L:D cycle).<br>For the other organisms, we used publicly available sequence data (see "Data collection" below).
Sampling strategy	We tried to collect new isolates from different locations.
Data collection	DNA (all strains, except Triparma laevis f. inornata) and RNA (for Triparma laevis f. inornate, Triparma strigata, Triparma retinervis and 'Scaly parma') were extracted using the DNeasy Plant Mini Kit or RNeasy Plant Mini Kit (Qiagen, Venlo, Netherlands), respectively. Libraries were generated using the Illumina TruSeq DNA/RNA sample preparation kit (Illumina, Inc., San Diego, USA). Sequencing of whole genomes or transcriptomes was performed on an Illumina HiSeq X (150 bp, paired-end) or HiSeq 2000 (100 bp, paired-end), respectively. Exceptionally, the genomes of Triparma laevis f. longispina and 'Scaly parma' were sequenced with an

Illumina HiSeq 2500 (150 bp, paired-end). DNA extraction and sequencing methods for Triparma laevis f. inornate were already reported in Kuwata et al., 2020.
For other organisms, we used publicly aaaavailable genomics and transcritomics data collected by previous studies.
Genomes:
Thalassiosira pseudonana: Armbrust et al., 2004
Thalassiosira oceanica: Lommer et al., 2012
Fragilariopsis cylindrus: Mock et al., 2017
Phaeodactylum tricornutum: Bowler et al., 2008
Pseudonitzschia multiseries: from JGI
Aureococcus anophagefferens: Gobler et al., 2011
Ectocarpus siliculosus: Cock et al., 2010
Phytophthora infestans: Haas et al., 2009
Phytophthora sojae: Tyler et al., 2006
Saprolegnia parasitica: Jiang et al., 2013

Transcriptomes:
MMETSP database: Keeling et al., 2014
Triparma pacifica RCC205: Kessenich et al., 2014

Timing and spatial scale	No time series data is used in our study.
Data exclusions	For orthologous genes clustering, we only use a part of ochrophyte transcriptomes (Supplementary Data 14) from the MMETSP database (Keeling et al., 2014) to reduce computation time.
Reproducibility	NA
Randomization	Randomization was not relevant to this study. The samples treated in this study (diatoms and parmaleans) cover a wide range of each phylogeny.
Blinding	Not relevant to this study because there are no situations that require blinding.

Did the study involve field work? ☐ Yes ☒ No

Reporting for specific materials, systems and methods

We require information from authors about some types of materials, experimental systems and methods used in many studies. Here, indicate whether each material, system or method listed is relevant to your study. If you are not sure if a list item applies to your research, read the appropriate section before selecting a response.

Materials & experimental systems

n/a	Involved in the study
☒	☐ Antibodies
☒	☐ Eukaryotic cell lines
☒	☐ Palaeontology and archaeology
☒	☐ Animals and other organisms
☒	☐ Clinical data
☒	☐ Dual use research of concern

Methods

n/a	Involved in the study
☒	☐ ChIP-seq
☒	☐ Flow cytometry
☒	☐ MRI-based neuroimaging
